# Effects of Yoga Interventions on Health Outcomes in Women With Gynecological Cancers: A Systematic Review of Randomised Controlled Trials

**DOI:** 10.7759/cureus.95017

**Published:** 2025-10-20

**Authors:** Selvaraj Giridharan, Nandan M Shanbhag, Abdulrahman Bin Sumaida, Khalid Balaraj

**Affiliations:** 1 Medical Oncology, Tawam Hospital, Al Ain, ARE; 2 Radiation Oncology, Tawam Hospital, Al Ain, ARE

**Keywords:** gynaecological cancers, psychological well-being, quality of life, systematic review, yoga

## Abstract

Gynaecological cancers have a profound impact on women's health, with survivors often experiencing a diminished quality of life (QoL) owing to treatment-related symptoms such as fatigue, psychological distress, and cognitive impairment. Yoga, as a mind-body intervention, may offer supportive benefits, although evidence specific to this population remains limited. This systematic review synthesised randomised controlled trials (RCTs) that evaluated the effects of yoga on health outcomes in women with gynaecological cancers. In accordance with the Preferred Reporting Items for Systematic Reviews and Meta-Analyses (PRISMA) guidelines, databases (PubMed, Scopus, Web of Science, Cochrane, and Google Scholar) were searched from inception to June 2025. Eligible RCTs included those involving adult women with gynaecological cancers, yoga interventions lasting ≥4 weeks, and outcomes such as QoL, psychological well-being, fatigue, and cognition. Risk of bias was assessed using RoB 2 (Cochrane, London, United Kingdom) (some concerns overall), and a narrative synthesis was conducted owing to heterogeneity. Grading of Recommendations Assessment, Development and Evaluation (GRADE) was used to assess the certainty of the primary outcomes. Six RCTs comprising 320 participants were included. Meditative yoga (for example, yoga nidra and pranayama) reduced anxiety and depression (P = 0.00001-0.026; narrative standardized mean difference (SMD) -0.56 (95%CI -1.01 to -0.11) for depression, GRADE: moderate) and stress (P<0.0001) in four trials. QoL improved in three trials (P=0.039-<0.05; narrative SMD ~0.3 (95% CI 0.12-0.48), GRADE: low), fatigue in two (P=0.039-<0.05; narrative SMD -1.17 (95%CI -2.16 to -0.18), GRADE: low), and cognitive function in one pilot (P=0.0007 for fluid cognition; Cohen's d=0.6, GRADE: very low). Restorative yoga showed higher adherence than vigorous forms. No adverse events were observed. Preliminary evidence suggests that yoga may enhance psychological well-being, QoL, fatigue, and cognition, which is consistent with broader oncology reviews. Larger, standardised RCTs are necessary to confirm the efficacy of this treatment and inform its integration into supportive care.

## Introduction and background

Gynaecological cancers, including malignancies of the cervix, ovary, uterus, vagina, and vulva, pose a significant global health challenge. According to the latest estimates from the Global Cancer Observatory, these cancers collectively account for approximately 1.4 million new cases and 680,000 deaths worldwide [1]. Cervical cancer alone contributed to 662,301 incident cases and 348,874 deaths, ranking as the fourth most common cancer among women globally, with over 85% of cases occurring in low- and middle-income countries (LMICs) [2]. In contrast, ovarian cancer, often diagnosed at an advanced stage due to vague early symptoms and lack of screening, remains the most lethal gynaecological cancer, with 324,603 new cases and 206,956 deaths reported in 2022 [3]. Endometrial cancer, which is increasingly linked to obesity and metabolic syndrome, accounted for 420,368 new cases globally [4]. Although vulvar and vaginal cancers are relatively rare, with approximately 47,000 new cases and 19,000 deaths from vulvar cancer and approximately 23,000 new cases and 9,000 deaths from vaginal cancer, they contribute disproportionately to morbidity, particularly in LMICs, where access to screening and vaccination remains limited [5]. Survival rates vary considerably according to cancer type and region. The global five-year net survival rate for cervical cancer is approximately 66%, dropping below 50% in LMICs, while ovarian cancer survival hovers around 45-51%, with recurrence rates exceeding 70% in advanced stages [6]. Endometrial cancer generally has a more favourable prognosis (five-year survival >80% in high-income countries), although outcomes remain poor for advanced or high-risk histologies [6,7]. Beyond mortality, treatment-related morbidities, such as infertility, lymphoedema, neurotoxicity, and premature menopause, affect millions of survivors globally, contributing to long-term reductions in QoL [8].

Standard treatment modalities for gynaecological cancers, including surgery, chemotherapy, radiotherapy, and, where appropriate, targeted therapies and immunotherapies, have contributed to significant improvements in survival outcomes [9,10]. However, these interventions are frequently accompanied by persistent side effects that can markedly diminish QoL. Women undergoing treatment often experience cancer-related fatigue, chronic pain, premature or treatment-induced menopause, psychological distress, sexual dysfunction, and sleep disturbances [11]. Such complications not only affect daily functioning but also impair the long-term survival of the patients. The additional burden of infertility and changes in body image further compound these challenges, underscoring the complex interplay between physical and psychological sequelae [12]. These persistent symptoms highlight the need for accessible, non-pharmacological interventions like yoga to support holistic survivorship. Yoga, unlike general relaxation or exercise, integrates mindful movement and breathing to promote parasympathetic activation and stress reduction

Despite survivorship guidelines from major oncology organisations emphasising comprehensive symptom management, many women continue to report unmet supportive care needs [13]. Access to evidence-based complementary and integrative interventions remains inconsistent, especially in resource-limited settings, including cultural and logistical barriers to practices such as yoga in LMICs. With rising survival rates, especially for early-stage cervical and endometrial cancers, the demand for holistic, integrative strategies to improve survivorship has grown more urgent [14]. These challenges highlight the importance of accessible interventions like yoga.

Yoga is a multifaceted mind-body discipline originating in India, encompassing physical postures (asanas), breathing techniques (pranayama), meditation and mindfulness (dhyana), and relaxation practices [15]. Over the past two decades, yoga has gained traction in oncology as a supportive intervention for cancer patients. Multiple systematic reviews and meta-analyses have demonstrated the benefits of exercise in diverse cancer populations, particularly in breast and prostate cancer survivors [16-18]. Reported outcomes include reductions in fatigue, anxiety, depression, and sleep disturbances, as well as improvements in overall QoL and functional well-being [19,20]. The proposed mechanisms underpinning these benefits include neuroendocrine modulation (e.g., reduced cortisol and sympathetic activity), immune regulation (e.g., enhanced natural killer cell activity and lower pro-inflammatory cytokines), psychological adaptation (e.g., improved coping and mindfulness), and physiological improvements in cardiorespiratory fitness and autonomic balance [21-23]. Emerging research also suggests the influence of the gut microbiome and epigenetic modifications, potentially enhancing the anti-cancer effects and reducing inflammation in patients with cancer [24,25]. These mechanistic pathways align with the stress-related allostatic load observed in patients with cancer, positioning yoga as a potentially valuable adjunctive therapy in oncology.

Building on this foundation, research tailored to gynaecological cancers is emerging. Emerging evidence from pilot studies and RCTs suggests the potential benefits of yoga [26-28]. Preliminary outcomes from these studies include enhanced QoL, reduced fatigue, improved mood, better coping mechanisms, and decreased distress, with adherence rates often exceeding 80%. However, gaps persist, such as the lack of large-scale RCTs, small sample sizes (often under 100 participants), heterogeneity in yoga styles (e.g., restorative versus vigorous), and limited mechanistic data specific to gynaecological populations. Given the significant burden of gynaecological cancers and the distinct challenges encountered by survivors, there is an imperative need to assess integrative approaches such as yoga [29-30]. Systematic reviews have extensively synthesised the evidence for yoga in oncology [31]. Recent reviews have examined mind-body therapies for gynaecological cancers [32]; however, none have specifically focused on yoga interventions within this population with rigorous synthesis. This systematic review addresses these gaps by synthesising evidence from clinical trials of yoga interventions in women with gynaecological cancer. By focusing on this understudied group, where treatment uniquely impacts reproductive and pelvic health, this review underscores the potential of evidence-based integrative approaches to enhance survivorship. Ultimately, it aims to inform clinical practice and future research, advocating for yoga as a safe and accessible tool to alleviate distress and improve quality of life in this vulnerable population.

## Review

Methods

This systematic review was conducted in accordance with the Preferred Reporting Items for Systematic Reviews and Meta-Analyses (PRISMA) 2020 guidelines [33]. The study included randomised controlled trials (RCTs) that were identified through a comprehensive search of databases, including PubMed, Scopus, Web of Science, Google Scholar, and Cochrane, from their inception to June 2025. The search strategy combined Medical Subject Headings (MeSH) terms and free-text keywords related to the population, intervention, and outcomes, with no initial language or date restrictions applied. The detailed search strategy is given in the Appendices. Non-English records were excluded during the screening process. The protocol was not prospectively registered on PROSPERO, as the review was conceived in an ad hoc manner to explore emerging evidence, rather than as a predefined systematic review.

Eligibility Criteria

Studies were eligible for inclusion if they met the following criteria using the PICOS (Population, Intervention, Comparison, Outcome, and Study design) framework [34]. The population consisted of adult women aged 18 years or older diagnosed with gynaecological cancers, including cervical, ovarian, endometrial, uterine, vulvar, or vaginal cancer, at any stage (I-IV). Participants could be undergoing active treatment, such as surgery, chemotherapy, or radiotherapy, or be in the post-treatment survivorship phase. Studies with mixed cancer cohorts were included only if the gynaecological cancer subgroups were separately analysable or comprised at least 50% of the sample.

The study design was limited to RCTs, including pilot RCTs. Non-randomised studies, case reports, qualitative studies, reviews, and protocols were excluded. Additionally, studies in languages other than English, interventions combining yoga with other modalities without isolating yoga’s effects, studies involving paediatric or male populations, non-gynaecological cancers, and those with fewer than 20 participants were excluded to minimize bias from very small samples.

Intervention

Yoga interventions (meditative: e.g., yoga nidra, pranayama; physical: e.g., restorative/vigorous hatha; music-based: e.g., nada yoga), including components like breathing exercises, relaxation, and gentle movements, delivered over ≥4 weeks. Interventions could be supervised, home-based, or delivered digitally, with a minimum duration of four weeks to allow for meaningful outcome assessment. The comparator included standard care, waitlist control, or active comparators such as other exercise or psychological interventions.

The primary outcomes assessed were quality of life (QoL) using the European Organisation for Research and Treatment of Cancer Quality of Life Questionnaire Core 30 (EORTC QLQ C30), psychological well-being (anxiety, depression, and stress) using the Hospital Anxiety and Depression Scale (HADS), Hamilton Anxiety Rating Scale (HAM-A), and Perceived Stress Scale (PSS-10) respectively, and fatigue. Secondary outcomes included physical and cognitive function (e.g., National Institutes of Health (NIH) Toolbox for the Assessment of Neurological and Behavioral Function), treatment-related symptoms (e.g., nausea and dyspnoea), feasibility (e.g., adherence rates), and safety (e.g., adverse events). Studies were selected if they reported at least one primary outcome.

Two reviewers independently screened titles, abstracts, and full texts using Rayyan software, resolving disagreements by consensus or with a third reviewer [35]. The risk of bias was assessed using the Cochrane Risk of Bias 2 (RoB 2) tool for RCTs and pilot studies [36]. Inter-rater agreement for risk-of-bias assessments was high (Cohen’s κ=0.85), with discrepancies resolved by consensus.

No meta-analysis was conducted due to heterogeneity; effect sizes and P-values are reported directly from original studies to avoid pooling errors. A narrative synthesis was performed, structured by outcome domains [37]. Findings were grouped by intervention type (e.g., meditative versus vigorous yoga) and patient population (active treatment versus survivorship). Effect sizes, such as Cohen’s d, were calculated from reported means and standard deviations where available. Heterogeneity was qualitatively described, and subgroup patterns (e.g., by yoga style) were explored narratively. The certainty of evidence was evaluated using the Grading of Recommendations Assessment, Development, and Evaluation (GRADE) approach [38].

Results

Study Selection

**Figure 1:**
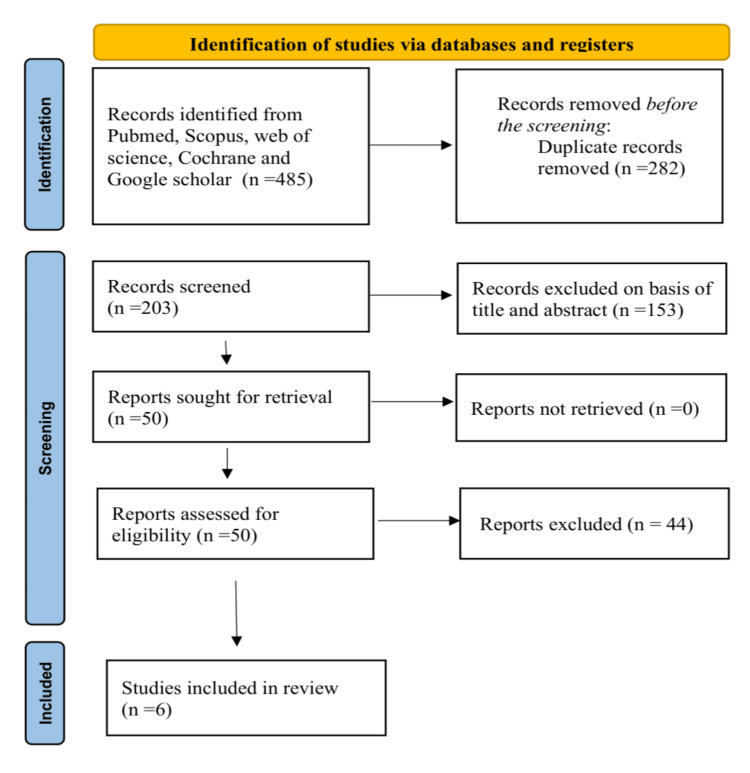
PRISMA flow diagram showing the summarized search strategy PRISMA: Preferred Reporting Items for Systematic Reviews and Meta-Analyses

A thorough systematic literature search initially identified 485 records. After removing 282 duplicates, 203 records were reviewed. Of these, 153 were considered irrelevant based on their title and abstract. A full-text review of 50 articles led to the exclusion of 44 for the following reasons: 29 were excluded due to non-relevant interventions, 10 for non-gynaecological cancers, and five for non-clinical trial designs. Consequently, six clinical trials were included in this systematic review [39-44]. The PRISMA flow diagram is presented in Figure 1. Aggregate protocols typically involved 15-60 minute sessions, three to seven days/week, emphasizing mindfulness, breathing, and tailored adaptations for cancer patients.

Characteristics of the Included Studies

The six trials included were all randomized controlled trials, with sample sizes ranging from 35 to 83 female participants, with a total of 320 participants, focusing on gynaecological cancers. Specifically, three trials addressed cervical cancer [39,40,44], one trial focused on ovarian cancer [43], and two trials examined mixed breast/ovarian cancer [41,42]. Cancer stages varied from I to IVA, with participants either undergoing treatment (chemotherapy or radiotherapy) or in the post-treatment survivorship phase of their disease.

The interventions included meditative practices like yoga nidra (audio-guided relaxation, 23 minutes daily during radiotherapy) [39], and pranayama combined with yoga nidra (30 minutes twice daily, five days/week for six weeks [40], focusing on breathing and relaxation); physical yoga, including restorative (gentle movements, meditative focus) versus vigorous (aerobic hatha) yoga (60 minutes three times/week for 12 weeks supervised, followed by 12 weeks home practice) [41,42]; music-based nada yoga therapy (15 minutes daily for 12 weeks, with or without verbal meditation instructions, delivered via a mobile app) [43]; and combined yoga with psychological intervention (gentle yoga with cognitive-behavioral elements during chemotherapy cycles, assessed at second, fourth, and sixth sessions) [44].

These protocols varied in delivery (supervised, audio-guided, or digital) but typically included relaxation, breathing, and mindfulness components tailored for cancer patients. The control groups received standard care in four trials, whereas two trials compared active yoga arms without a no-intervention control. The trial durations ranged from five to 24 weeks, with outcomes assessed at baseline and post-intervention. Primary outcomes included psychological well-being (anxiety, depression, stress via HADS, HAM-A, PSS-10), quality of life (EORTC QLQ-C30, SF-36), cognitive function (NIH Toolbox), fatigue, and feasibility/adherence. The characteristics of the included studies are summarised in Table 1.

**Table 1:** Characteristics of Included Studies NR: not reported; HADS: Hospital Anxiety and Depression Scale; PSS-10: Perceived Stress Scale; EORTC QLQ-C30: European Organization for Research and Treatment of Cancer Quality of Life Questionnaire; HAM-A: Hamilton Anxiety Rating Scale; CRF: Cancer-Related Fatigue; SF-36: 36-Item Short Form Survey; RCT: randomised controlled trial

Study	Study Design	Sample Size	Cancer Type	Stage	Country	Intervention Details	Control Group	Duration	Frequency	Outcomes Assessed
D’cunha et al. [39]	RCT	48	Cervical	I-III	India	Yoga Nidra, 23 min daily audio-guided	Standard care	5 weeks	5 days/week	Stress (PSS-10)
Nuzhath et al. [40]	RCT	70	Cervical	I-IVA	India	Pranayama + Yoga Nidra, 30 min twice daily	Standard care	6 weeks	5 days/week	Anxiety, Depression (HADS)
Lapen et al. [41]	Pilot RCT	35	Breast/Ovarian	0-III	United States	Restorative vs. Vigorous Yoga, 60 min	No control	24 weeks	3 sessions/week (12 supervised, 12 home)	Feasibility, Adherence
Deng et al. [42]	Pilot RCT	35	Breast/Ovarian	0-III	United States	Restorative vs. Vigorous Yoga, 60 min	No control	24 weeks	3 sessions/week (12 supervised, 12 home)	Cognitive Function (NIH Toolbox)
Malik et al. [43]	RCT	49	Ovarian	NR	India	Nada Yoga Music, 15 min daily (with/without meditation)	Active control (other arm)	12 weeks	Daily	Anxiety (HAM-A), QoL (EORTC QLQ-C30)
Yang et al. [44]	RCT	83	Cervical	NR	China	Combined yoga + psychological intervention during chemo	Standard care	~12 weeks (across 6 chemo cycles)	During chemo sessions	Fatigue (CRF scale), QoL (SF-36), Anxiety/Depression, Psych status

Risk of Bias/Quality Assessment

**Figure 2:**
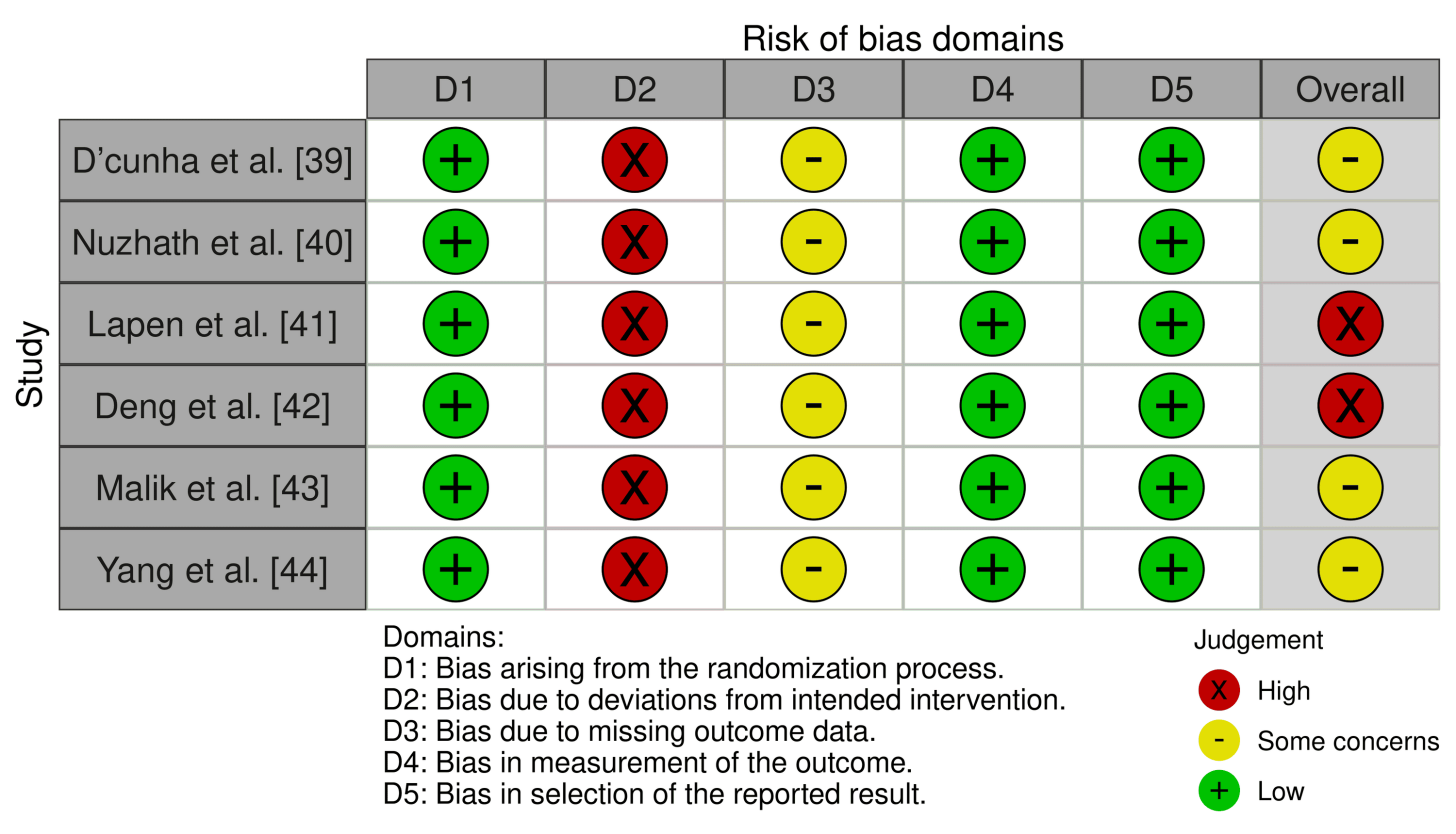
Risk of Bias Assessment Using the RoB 2 Tool Across Included Studies References: [39-44]

**Figure 3:**
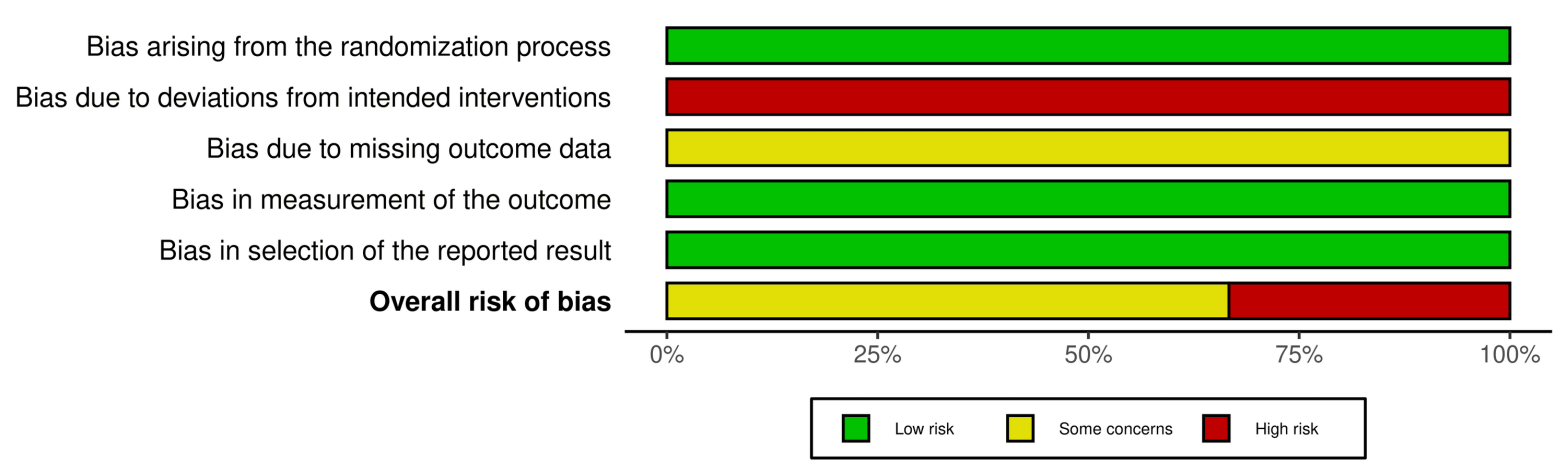
Summary Plot of Risk of Bias Assessments

The methodological quality of the six trials was moderate, with potential risks of bias primarily due to blinding and allocation concealment. None of the trials blinded the participants or personnel owing to the nature of the yoga intervention, which introduced performance bias. Detection bias was possible in self-reported outcomes (e.g., anxiety and stress). Randomisation was adequately described in all trials (e.g., lottery method, computer-generated), but allocation concealment was unclear in three trials. Attrition was low (0-15%), with reported reasons (e.g., unrelated medical issues). Selective reporting was low because all prespecified outcomes were addressed. Overall, four trials had a moderate risk of bias, and two pilot trials had a higher risk of bias owing to their small sample sizes and feasibility focus. The traffic light plot and summary plot of the risk of bias assessment using the RoB 2 tool across included studies are presented in Figure 2 and Figure 3, respectively.

The certainty of evidence was rated using the GRADE approach, starting from high for RCTs and downgraded for risk of bias, inconsistency, indirectness, and imprecision of the evidence. Overall, evidence for psychological well-being was moderate, while QoL, fatigue, and cognitive function were low to very low owing to heterogeneity and small sample sizes. The Grade Table indicates moderate certainty for psychological well-being but low-to-very low for QoL and fatigue, driven by inconsistency and imprecision (Table 2).

**Table 2:** GRADE Summary of Findings GRADE ratings: High (further research unlikely to change confidence); Moderate (may change with new evidence); Low (further research likely to change); Very Low (very uncertain). No publication bias assessed (few studies). GRADE: Grading of Recommendations Assessment, Development and Evaluation; RCT: randomised controlled trial; SMD: standardized mean difference

Outcome	No. of Studies (Participants)	Summary of Findings (Effect Estimate)	Quality of Evidence (GRADE)	Rationale for GRADE Rating
Quality of Life	3 (201)	Moderate improvements in global health status and SF-36 scores (P=0.039 to <0.05; narrative SMD ~0.3-0.5 favoring yoga). Consistent trends but heterogeneous measures.	Low	High start (RCTs); Downgraded 2 levels: Inconsistency (high heterogeneity in tools/durations); Imprecision (small n, no pooled CI).
Psychological Well-Being (Anxiety/Depression/Stress)	5 (248)	Significant reductions (P<0.00001 to 0.026; narrative SMD -0.56 to -1.17 favoring meditative yoga). Consistent across meditative interventions.	Moderate	High start (RCTs); Downgraded 1 level: Inconsistency (moderate, due to varying scales); Offset by consistency in direction.
Fatigue	2 (132)	Significant reductions (P=0.039 to <0.05; narrative SMD -1.17 to -1.60 favoring yoga). Limited but consistent.	Low	High start (RCTs); Downgraded 2 levels: Inconsistency (few studies, heterogeneous chemo contexts); Imprecision (small n).
Cognitive Function	1 (35)	Improvements in fluid cognition with restorative yoga (P=0.0007; Cohen's d=0.6 vs. vigorous). Pilot-level evidence.	Very Low	High start (RCT); Downgraded 3 levels: Risk of bias (high in pilot); Inconsistency (single study); Imprecision (very small n=35).
Physical Functioning/Treatment-Related Symptoms	1 (49)	No change in physical function (P=0.390); improvements in nausea/dyspnea (P=0.009-0.011). Inconsistent.	Very Low	High start (RCT); Downgraded 3 levels: Inconsistency (limited data, mixed symptoms); Imprecision (small n); Indirectness (chemotherapy-specific).

Effects of Yoga Interventions

Due to the diverse nature of the interventions, outcomes, and populations, a meta-analysis was not feasible; instead, the results were conveyed through a narrative synthesis. Table 3 provides a summary of the effects of yoga interventions, categorised by outcome domains.

**Table 3:** Effects of Yoga Interventions by Outcome Domain P-values reflect original trial reports; between-group P-values where available; cognitive data reported as significant P-values due to lack of exact means in summary. NR: not reported; SD: standard deviation; NS: not significant; SF-36 QoL: 36-Item Short Form Health Survey; PSS-10: Perceived Stress Scale-10; HADS: Hospital Anxiety and Depression Scale; CRF: Cancer-Related Fatigue

Outcome Domain	Study Author, Year	Intervention Group (n)	Baseline Mean (SD)	Post-Intervention Mean (SD)	Control/Comparison Group (n)	Baseline Mean (SD)	Post-Intervention Mean (SD)	P-Value (Within-Group)	P-Value (Between-Group)
Global Health Status	Malik et al., 2024 [43]	Group A (25)	60.07 (NR)	77.78 (NR)	Group B (24)	65.33 (NR)	70.00 (NR)	NR	0.039
SF-36 QoL	Yang et al., 2021 [44]	Observation (45)	NR	Improved (P<0.05)	Control (38)	NR	No change	<0.05	<0.05
Physical Functioning	Malik et al., 2024 [43]	Group A (25)	NR	NR	Group B (24)	NR	NR	NR	0.390
Anxiety (HADS)	Nuzhath et al., 2024 [40]	Experimental (35)	8.2 (0.95)	6.2 (1.71)	Control (35)	8.2 (0.97)	11.1 (1.29)	<0.00001	<0.00001
Depression (HADS)	Nuzhath et al., 2024 [40]	Experimental (35)	7.94 (1.16)	7.11 (1.75)	Control (35)	8.4 (1.11)	12.2 (1.29)	<0.00001	<0.00001
Stress (PSS-10)	D’cunha et al., 2021 [39]	Experimental (24)	80.21 (4.32)	64.42 (3.28)	Control (24)	81.04 (5.01)	79.46 (5.63)	<0.0001	<0.0001
Anxiety (HAM-A)	Malik et al., 2024 [43]	Group A (25)	11.67 (4.18)	6.79 (2.75)	Group B (24)	9.28 (3.05)	9.32 (4.68)	NR	0.026
Anxiety/Depression	Yang et al., 2021 [44]	Observation (45)	NR	Lower post-4th/6th (P<0.05)	Control (38)	NR	Higher	<0.05	<0.05
Fatigue	Malik et al., 2024 [43]	Group A (25)	36.11 (NR)	28.24 (NR)	Group B (24)	22.67 (NR)	37.33 (NR)	NR	0.039
Total Fatigue (CRF)	Yang et al., 2021 [44]	Observation (45)	11.28 (4.14)	16.39 (6.33)	Control (38)	12.76 (4.63)	18.84 (5.65)	<0.05	<0.05
Physical Functioning	Malik et al., 2024 [43]	Group A (25)	NR	NR	Group B (24)	NR	NR	NR	0.390
Nausea/Vomiting	Malik et al., 2024 [43]	Group A (25)	NR	NR	Group B (24)	NR	NR	NR	0.009
Dyspnea	Malik et al., 2024 [43]	Group A (25)	NR	NR	Group B (24)	NR	NR	NR	0.011
Overall Cognition	Deng et al., 2022 [42]	Restorative (17)	116 (10.3)	Improved (P=0.03)	Vigorous (18)	115.5 (11.1)	No change	0.03 (week 12)	NR
Fluid Cognition	Deng et al., 2022 [42]	Restorative (17)	97.1 (8.3)	Improved (P=0.02)	Vigorous (18)	95.2 (7)	No change	0.02 (week 12)	NR (week 24 diff)
Crystallized Cognition	Deng et al., 2022 [42]	Restorative (17)	128.7 (10.7)	No change	Vigorous (18)	130.2 (9.6)	Improved (P=0.03)	NS	NR

Quality of Life

Two trials assessed QoL, showing consistent moderate improvements with meditative and combined interventions [43,44]. In the nada yoga trial (n=49 ovarian cancer patients), Group A (music without instructions) significantly improved global health status (baseline 60.07 to post 77.78) versus Group B (65.33 to 70.00; between-group P=0.039), though functional scales (physical, role, emotional, cognitive, social) showed no changes (P>0.05) [43]. Similarly, the combined yoga and psychological intervention trial (n=83 cervical cancer patients) reported better SF-36 scores in the observation group post-intervention (e.g., overall QoL and dimensions like mental health/vitality improved from baseline; P<0.05) [44].

Psychological Well-Being

Four trials evaluated psychological outcomes [39,40,43,44]. In the pranayama + yoga nidra trial (n=70 cervical cancer patients), the experimental group showed significant reductions in anxiety (baseline mean 8.2±0.95 to week 6:6.2±1.71; P<0.00001) and depression (7.94±1.16 to 7.11±1.75; P<0.00001) compared to controls (anxiety: 8.2±0.97 to 11.1±1.29; depression: 8.4±1.11 to 12.2±1.29) [40]. In the yoga nidra trial (n=48 cervical cancer patients), stress was significantly reduced in the experimental group (baseline 80.21±4.32 to post: 64.42±3.28; P<0.0001) versus controls (81.04±5.01 to 79.46±5.63) [39]. The nada yoga trial reported lower anxiety in Group A (baseline 11.67±4.18 to post: 6.79±2.75) versus Group B (9.28±3.05 to 9.32±4.68; P=0.026) [43]. In a combined yoga and psychological intervention trial (n=83), the observation group had lower anxiety/depression scores post-4th/6th chemo (e.g., anxiety: 36.72±6.44 vs. control 43.53±5.57; P<0.05) [44]. The findings were consistent with reductions in anxiety, depression, and stress with meditative or combined yoga forms.

Fatigue

Two trials reported fatigue [43,44]. In the nada yoga trial (n=49), significant fatigue reduction was observed in Group A (baseline mean 36.11 to post: 28.24) versus Group B (22.67 to 37.33; P=0.039) [43]. In a combined yoga and psychological intervention trial (n=83), the observation group had lower total fatigue during/after chemotherapy (for example, 16.39±6.33 vs. control 18.84±5.65 post-chemotherapy; P<0.05) [44].

Physical Functioning

One trial showed no significant changes in physical functioning (P=0.390) or other symptom scales, such as pain (P=0.534), insomnia (P=0.309), appetite loss (P=0.270), constipation (P=0.320), or diarrhoea (P=0.070). Nausea/vomiting (P=0.009) and dyspnea (P=0.011) improved in Group A [43].

Treatment-Related Symptoms

The nada yoga trial reported improvements in nausea/vomiting, dyspnea, and financial difficulties (P<0.05) in Group A [43]. The combined yoga trial noted reduced fatigue and improved mental health [44]. Consistency was observed for symptom relief with music-based or combined yoga, but limited data precluded broader conclusions from being drawn. Heterogeneity was high across trials in terms of yoga type, duration, and outcome, limiting direct comparisons.

Subgroup/Intervention Characteristics

Meditation-focused interventions showed patterns of greater benefits for psychological outcomes than physical-focused vigorous yoga [39,40,43]. Restorative yoga is associated with better adherence and cognitive improvements than vigorous yoga [41,42]. Combining yoga with psychological intervention targeted fatigue and QoL during chemotherapy [44]. Shorter durations (5-6 weeks) targeted acute treatment stress, while longer (12-24 weeks) addressed survivorship. Patients undergoing active treatment (cervical/ovarian) benefited from relaxation-based yoga, while survivors (breast/ovarian) showed feasibility for supervised programs.

Discussion

Summary of Findings

Across the six included trials, yoga interventions demonstrated trends toward improvements in psychological well-being, quality of life, fatigue, and cognitive function in women with gynaecological cancers. Specifically, meditation-focused yoga (for example, yoga nidra, pranayama + yoga nidra, and nada yoga music) consistently reduced anxiety, depression, and stress in active treatment populations, with significant p-values (<0.00001 to 0.026) across four trials [39,40,43,44]. QoL improvements were observed in three trials, with global health status and SF-36 scores showing moderate to large effects (P=0.039 to <0.05), particularly in meditative and combined interventions during chemotherapy [43,33]. Fatigue reduction was reported in two trials, with significant between-group differences (P=0.039 to <0.05) favouring the yoga group. Cognitive function benefits were noted in one pilot RCT, with restorative yoga improving fluid cognition (P=0.0007) over vigorous yoga [42]. Treatment-related symptoms, such as nausea/vomiting and dyspnoea, improved in one trial (P=0.009 to 0.011) [43]. No major adverse events were observed.

Heterogeneity was evident in yoga types (meditative vs. vigorous/combined), durations (5-24 weeks), and outcomes, limiting generalisations. Meditative interventions appeared more beneficial for psychological outcomes in active treatment, while restorative yoga showed higher adherence to survivorship. The risk of bias was moderate overall, driven by the lack of blinding and unclear allocation concealment in half of the trials; pilot studies had a higher risk due to small sample sizes.

Comparison with Previous Reviews

These findings are consistent with those of broader oncology reviews [31,32]. Niu et al. synthesised 34 RCTs on yoga for cancer survivors, reporting small-to-moderate effects on physical function (SMD 0.28), mental health (SMD -0.51 for fatigue), and QoL (SMD 0.28), primarily in breast cancer cohorts [31]. The trends in fatigue reduction (SMD-1.17 to-1.60) and QoL improvements in the current review align, although the effects on psychological well-being appear larger here (SMD -0.56 to -1.17), possibly due to gynaecological-specific stressors such as pelvic symptoms. However, Niu et al. noted high heterogeneity (I² >50% in many analyses), mirroring the present synthesis, in which meta-analysis was precluded.

Ong et al. reviewed mind-body therapies (including yoga) in women with gynaecological cancers across nine RCTs, finding significant effects on depression (SMD -0.56), pain (SMD -1.60), and fatigue (SMD -1.17), but not QoL or anxiety [32]. These estimates closely match current yoga-specific findings, suggesting that yoga contributes substantially to mind-body benefits. Differences include Ong et al.'s broader scope (e.g., including qigong and meditation) [32], which may explain the non-significant QoL effects, while the yoga-focused trials here showed moderate improvements in global health status. Both reviews highlighted low evidence quality due to bias risks and heterogeneity, with Ong et al. emphasising gynaecological underrepresentation in mind-body research, consistent with the limited trials identified here [32].

Future Directions

Gaps persist in small samples, heterogeneous interventions, and the absence of mechanistic outcomes (e.g., immune/endocrine markers). The limited long-term follow-up restricts insights into survivorship. Priorities for future research include larger multicentre RCTs with standardised protocols to enable meta-analyses. A longer follow-up (≥6 months) is needed to determine the enduring effects on QoL and symptomatology. Incorporating biological markers (e.g., cortisol and cytokines) could elucidate these mechanisms. Comparative effectiveness trials (yoga vs. other mind-body therapies, such as mindfulness) would clarify the optimal approach. The emerging themes included co-created programs. Price et al. developed a 12-week hatha yoga program for gynaecologic cancer via consensus, emphasising bio-psycho-social needs and feasibility (online/in-person) [45]. Feasibility trials of tailored hatha yoga show promise for fatigue and distress reduction, warranting efficacy testing in larger samples. These gaps underscore the translational potential of integrating yoga into guidelines to enhance supportive care, particularly in underserved gynaecological populations. Potential psychosocial confounders, such as group-based social interaction or concurrent therapies, may contribute to psychological improvements and warrant control in future designs.

## Conclusions

This systematic review provides evidence that yoga interventions positively influence health outcomes in women with gynaecological cancers, particularly psychological well-being, quality of life, fatigue, and cognitive function. Meditative forms such as yoga nidra and pranayama alleviated anxiety, depression, and stress during treatment, while restorative yoga enhanced cognition and adherence in survivorship. However, limitations include small sample sizes, heterogeneous protocols, and moderate bias risks, precluding meta-analysis and causal inferences. The absence of mechanistic data and long-term follow-up constrains the conclusions. Future research should prioritise large-scale, multicentre RCTs with standardised interventions, incorporating biomarkers and comparative designs to elucidate the mechanisms and optimise the applications. These efforts may support the integration of yoga into survivorship guidelines, offering a safe adjunct to improve holistic well-being in this underserved population.

## Appendices

Detailed search strategies

The complete search strategies employed for this systematic review, specific to each database, are detailed below. Searches were conducted from database inception to June 30, 2025. Terms were combined using Boolean operators (AND/OR) and adapted to each database's syntax (e.g., MeSH terms for PubMed). No language or date filters were initially applied beyond the end date. The core strategy focused on: (1) yoga interventions (e.g., "yoga" OR "pranayama" OR "nidra"), (2) gynaecological cancers (e.g., "gynecological cancer" OR "cervical cancer" OR "ovarian cancer"), and (3) study design/outcomes (e.g., "randomized controlled trial" AND ("quality of life" OR "anxiety" OR "fatigue" OR "cognition")).

1. PubMed/MEDLINE

text

("yoga"[MeSH Terms] OR "yoga"[Title/Abstract] OR "hatha yoga"[Title/Abstract] OR "pranayama"[Title/Abstract] OR "yoga nidra"[Title/Abstract] OR "nada yoga"[Title/Abstract] OR "asana"[Title/Abstract] OR "dhyana"[Title/Abstract])

AND

("genital neoplasms, female"[MeSH Terms] OR "gynecologic neoplasm"[Title/Abstract] OR "gynaecological cancer"[Title/Abstract] OR "cervical cancer"[Title/Abstract] OR "ovarian cancer"[Title/Abstract] OR "endometrial cancer"[Title/Abstract] OR "uterine cancer"[Title/Abstract] OR "vulvar cancer"[Title/Abstract] OR "vaginal cancer"[Title/Abstract])

AND

("randomized controlled trial"[Publication Type] OR "randomized"[Title/Abstract] OR "RCT"[Title/Abstract])

AND

("quality of life"[MeSH Terms] OR "anxiety"[MeSH Terms] OR "depression"[MeSH Terms] OR "stress, psychological"[MeSH Terms] OR "fatigue"[MeSH Terms] OR "cognition"[MeSH Terms] OR "quality of life"[Title/Abstract] OR "anxiety"[Title/Abstract] OR "depression"[Title/Abstract] OR "stress"[Title/Abstract] OR "fatigue"[Title/Abstract] OR "cognitive function"[Title/Abstract])

2. Scopus

text

TITLE-ABS-KEY (yoga OR "hatha yoga" OR pranayama OR "yoga nidra" OR "nada yoga" OR asana OR dhyana)

AND

TITLE-ABS-KEY ("gynecological cancer" OR "gynaecological cancer" OR "cervical cancer" OR "ovarian cancer" OR "endometrial cancer" OR "uterine cancer" OR "vulvar cancer" OR "vaginal cancer")

AND

TITLE-ABS-KEY (randomized OR RCT OR "randomised controlled trial")

AND

TITLE-ABS-KEY ("quality of life" OR anxiety OR depression OR stress OR fatigue OR cognition OR "cognitive function")

3. Web of Science (Core Collection)

text

TS=((yoga OR "hatha yoga" OR pranayama OR "yoga nidra" OR "nada yoga" OR asana OR dhyana)

AND

("gynecological cancer" OR "gynaecological cancer" OR "cervical cancer" OR "ovarian cancer" OR "endometrial cancer" OR "uterine cancer" OR "vulvar cancer" OR "vaginal cancer")

AND

(randomized OR RCT OR "randomised controlled trial")

AND

("quality of life" OR anxiety OR depression OR stress OR fatigue OR cognition OR "cognitive function"))

4. Cochrane Library (CENTRAL)

text

"yoga" OR "hatha yoga" OR pranayama OR "yoga nidra" OR "nada yoga" OR asana OR dhyana

AND

"gynecological cancer" OR "gynaecological cancer" OR "cervical cancer" OR "ovarian cancer" OR "endometrial cancer" OR "uterine cancer" OR "vulvar cancer" OR "vaginal cancer"

AND

(randomized OR RCT OR "randomised controlled trial")

AND

"quality of life" OR anxiety OR depression OR stress OR fatigue OR cognition OR "cognitive function"

5. Google Scholar

text

"yoga" "gynaecological cancer" OR "gynecological cancer" OR "cervical cancer" OR "ovarian cancer" "randomized controlled trial" OR RCT "quality of life" OR anxiety OR fatigue OR cognition

## References

[1] Bray F, Laversanne M, Sung H, Ferlay J, Siegel RL, Soerjomataram I, Jemal A (2024). Global cancer statistics 2022: GLOBOCAN estimates of incidence and mortality worldwide for 36 cancers in 185 countries. CA Cancer J Clin.

[2] Arbyn M, Weiderpass E, Bruni L, de Sanjosé S, Saraiya M, Ferlay J, Bray F (2020). Estimates of incidence and mortality of cervical cancer in 2018: a worldwide analysis. Lancet Glob Health.

[3] Reid BM, Permuth JB, Sellers TA (2017). Epidemiology of ovarian cancer: a review. Cancer Biol Med.

[4] Lortet-Tieulent J, Ferlay J, Bray F, Jemal A (2018). International patterns and trends in endometrial cancer incidence, 1978-2013. J Natl Cancer Inst.

[5] Bray F, Laversanne M, Weiderpass E, Arbyn M (2020). Geographic and temporal variations in the incidence of vulvar and vaginal cancers. Int J Cancer.

[6] Matsuda T, Okuyama A (2018). Cancer incidence rates in the world from the Cancer Incidence in Five Continents XI. Jpn J Clin Oncol.

[7] Miller KD, Nogueira L, Devasia T (2022). Cancer treatment and survivorship statistics, 2022. CA Cancer J Clin.

[8] Wilson CM, McGuire DB, Rodgers BL, Elswick RK Jr, Temkin SM (2021). Body image, sexuality, and sexual functioning in women with gynecologic cancer: an integrative review of the literature and implications for research. Cancer Nurs.

[9] Tu M, Xu J (2023). Advances in immunotherapy for gynecological malignancies. Crit Rev Oncol Hematol.

[10] Silva E (2024). Cancer statistics, 2024: mixed results in gynecologic oncology. Int J Gynecol Cancer.

[11] Gil-Ibanez B, Davies-Oliveira J, Lopez G, Díaz-Feijoo B, Tejerizo-Garcia A, Sehouli J (2023). Impact of gynecological cancers on health-related quality of life: historical context, measurement instruments, and current knowledge. Int J Gynecol Cancer.

[12] Campbell G, Thomas TH, Hand L, Lee YJ, Taylor SE, Donovan HS (2019). Caring for survivors of gynecologic cancer: assessment and management of long-term and late effects. Semin Oncol Nurs.

[13] Dahl L, Wittrup I, Væggemose U, Petersen LK, Blaakaer J (2013). Life after gynecologic cancer--a review of patients quality of life, needs, and preferences in regard to follow-up. Int J Gynecol Cancer.

[14] Efficace F, Jacobs M, Pusic A (2014). Patient-reported outcomes in randomised controlled trials of gynaecological cancers: investigating methodological quality and impact on clinical decision-making. Eur J Cancer.

[15] Feuerstein G (2013). The Yoga Tradition: Its History, Literature, Philosophy and Practice. SCB Distributors.

[16] Danhauer SC, Addington EL, Sohl SJ, Chaoul A, Cohen L (2017). Review of yoga therapy during cancer treatment. Support Care Cancer.

[17] Danhauer SC, Addington EL, Cohen L, Sohl SJ, Van Puymbroeck M, Albinati NK, Culos-Reed SN (2019). Yoga for symptom management in oncology: a review of the evidence base and future directions for research. Cancer.

[18] Giridharan S, Soumian S, Kumar NV (2024). Yoga in cancer care: a bibliometric analysis of systematic reviews. Cureus.

[19] DiStasio SA (2008). Integrating yoga into cancer care. Clin J Oncol Nurs.

[20] Haier J, Duda A, Branss-Tallen C (2018). Improvement of well-being in cancer patients by yoga training. Med J Indon.

[21] Taso CJ, Lin HS, Lin WL, Chen SM, Huang WT, Chen SW (2014). The effect of yoga exercise on improving depression, anxiety, and fatigue in women with breast cancer: a randomized controlled trial. J Nurs Res.

[22] Kiecolt-Glaser JK, Bennett JM, Andridge R (2014). Yoga's impact on inflammation, mood, and fatigue in breast cancer survivors: a randomized controlled trial. J Clin Oncol.

[23] Mishra B, Agarwal A, George JA (2024). Effectiveness of yoga in modulating markers of immunity and inflammation: a systematic review and meta-analysis. Cureus.

[24] Blockhuys S, Wittung-Stafshede P (2024). Yoga as a complementary therapy for cancer patients: from clinical observations to biochemical mechanisms. Complement Med Res.

[25] Raman M, Vishnubhotla R, Ramay HR (2023). Isha yoga practices, vegan diet, and participation in Samyama meditation retreat: impact on the gut microbiome & metabolome - a non-randomized trial. BMC Complement Med Ther.

[26] Sohl SJ, Danhauer SC, Schnur JB, Daly L, Suslov K, Montgomery GH (2012). Feasibility of a brief yoga intervention during chemotherapy for persistent or recurrent ovarian cancer. Explore (NY).

[27] Danhauer SC, Tooze JA, Farmer DF, Campbell CR, McQuellon RP, Barrett R, Miller BE (2008). Restorative yoga for women with ovarian or breast cancer: findings from a pilot study. J Soc Integr Oncol.

[28] Lowe KA, Andersen MR, Sweet E, Standish L, Drescher CW, Goff BA (2012). The effect of regular exercise and yoga on health-related quality of life among ovarian cancer survivors. J Evid Based Complement Altern Med.

[29] Hanvey GA, Kacel EL, Bacharz KC (2024). Proof-of-concept of an integrated yoga and psychological intervention in mitigating distress among diverse women with gynecologic, gastrointestinal, and thoracic cancers. Integr Cancer Ther.

[30] Chase DM, Gibson SJ, Sumner DA, Bea JW, Alberts DS (2014). Appropriate use of complementary and alternative medicine approaches in gynecologic cancers. Curr Treat Options Oncol.

[31] Niu N, Huang R, Zhao J, Zeng Y (2024). Health benefits of yoga for cancer survivors: an updated systematic review and meta-analysis. Asia Pac J Oncol Nurs.

[32] Ong JW, Ong QO, Metsävainio T, Vaajoki A, Tian JL, He HG (2024). The effectiveness of mind-body therapies for women with gynecological cancer: a systematic review and meta-analysis. Cancer Nurs.

[33] Page MJ, McKenzie JE, Bossuyt PM (2021). The PRISMA 2020 statement: an updated guideline for reporting systematic reviews. BMJ.

[34] Richardson WS, Wilson MC, Nishikawa J, Hayward RS (1995). The well-built clinical question: a key to evidence-based decisions. ACP J Club.

[35] Ouzzani M, Hammady H, Fedorowicz Z, Elmagarmid A (2016). Rayyan-a web and mobile app for systematic reviews. Syst Rev.

[36] Higgins JP, Altman DG, Gøtzsche PC (2011). The Cochrane Collaboration's tool for assessing risk of bias in randomised trials. BMJ.

[37] Popay J, Roberts H, Sowden A (2006). Guidance on the Conduct of Narrative Synthesis in Systematic Reviews A Product from the ESRC Methods Programme. https://www.york.ac.uk/media/crd/Guidance%20on%20the%20conduct%20of%20narrative%20synthesis%20in%20systematic%20review.pdf.

[38] Neumann I, Santesso N, Akl EA (2016). A guide for health professionals to interpret and use recommendations in guidelines developed with the GRADE approach. J Clin Epidemiol.

[39] D’cunha R, Pappachan B, D’souza OL, Tonse R, Saldanha E, Baliga MS (2021). Effectiveness of yoga nidra in mitigating stress in women undergoing curative radiotherapy for cervical cancer. Middle East J Cancer.

[40] Nuzhath FJ, Patil NJ, Sheela SR, Manjunath GN (2024). A randomized controlled trial on pranayama and yoga nidra for anxiety and depression in patients with cervical cancer undergoing standard of care. Cureus.

[41] Lapen K, Benusis L, Pearson S (2018). A feasibility study of restorative yoga versus vigorous yoga intervention for sedentary breast and ovarian cancer survivors. Int J Yoga Therap.

[42] Deng G, Bao T, Ryan EL (2022). Effects of vigorous versus restorative yoga practice on objective cognition functions in sedentary breast and ovarian cancer survivors: a randomized controlled pilot trial. Integr Cancer Ther.

[43] Malik S, Sehrawat A, Chaturvedi J, Kumari R, Barnwal SL, Kalra S, Gupta S (2024). Impact of nada yoga music therapy on anxiety and quality of life in ovarian cancer patients: a randomized controlled trial. Int J Yoga.

[44] Yang H, Zhang Q, Liu L (2021). Effectiveness of combined yoga and psychological intervention on cancer-related fatigue in cervical cancer chemotherapy patients. Int J Clin Exp Med Res.

[45] Price J, Harris C, Praamsma N, Brunet J (2024). Co-creating a yoga program for women diagnosed with gynecologic cancer: a consensus study. Support Care Cancer.

